# Report of a Case of Purpura Hemorrhagica, with Remarks

**Published:** 1890-07

**Authors:** Charles D. Roy

**Affiliations:** Senior Assistant at Charity Hospital, New York


### REPORT OF A CASE OF PURPURA HEMORRHAGICA, WITH REMARKS.

BY CHARLES D. ROY, A. B., M. D.,
Senior Assistant at Charity Hospital, New York.

The name, Purpura Hemorrhagica, denotes a symptom of diverse pathological states rather than a distinctive disease with a well-defined etiology. That there are pathological conditions which occasion the symptoms in question, must be obvious to all, but they are so varied in their nature, and often so obscure as to baffle all endeavors at a thorough understanding of the same. Before going into the etiology of the disease, and into some explanatory remarks of the pathological condition, I will give the history of a case treated by me in the hospital. The patient, German, æt. 43, male, was admitted into the hospital on April 11th. A week previous to his entrance he had been discharged from the erysipelas pavilion, cured of one of the severest attacks of idiopathic facial erysipelas that I had ever seen. On again entering the hospital he asked to be transferred to the same pavilion, and though not a case for that locality, his wish, however, was gratified. I will remark at this juncture that the erysipelas pavilion is a distinct building, situated at the edge of the river, and thus isolated from the others, so as to cause no fear of contamination. From the patient I gained the following subjective history: He was employed upon a farm, where he worked continuously all day, and was compelled to stand most of the time. He had always been very healthy in his previous life, and had suffered with no disease except his last attack of erysipelas. For this latter he had been treated by the house surgeon who preceded me, being confined to the bed for two weeks. Says he was always a chronic bleeder, and would often bleed from the nose for half an hour at a time. On being discharged from the hospital, he again resumed his duties on the farm. On Thursday night preceding his entrance into the pavilion, which was the next day, he retired as usual, in excellent health. The next morning he felt his legs somewhat stiffened in his endeavor to rise from the bed, and on glancing down at his extremities he was surprised to find them covered with splotches of a purplish hue. Never having had such a condition before, he was somewhat alamed, and immediately started for the hospital. On entering the ward I found the following objective symptoms: His arms, but sparingly, from the centre of his body down, were covered with petechial spots, varying in size from that of millet seed to a ten cent coin. Most of them were discrete, while not a few were confluent. His face and chest were free from all manifestations of the disease. The lesions were purplish in color, of the sizes mentioned, and on running your finger over the surface, there was found to be no elevation of the same. In only three points were these distinct traces of hemorrhage into the skin with a marked elevation, and they were in the course of the long saphenous vein. The lesions were very numerous, around the buttocks and on the inner sides of the thighs. His hands and feet were somewhat swollen, but to no marked degree, while the veins showed very slight symptoms of varicosity. His temperature was taken, with the marking of 100 degrees, but no other febrile symptom. On examining a specimen of his urine the next morning, I found the color of it in close resemblance to a cherry red. The test for albumen resulted in nil, but on microscopic examination I found considerable mucus mixed with red blood corpuscles and an occasional pus cell. This showed there had evidently been a hemorrhage into the mucous membrane of the bladder. Cases of this kind are very rare, and I was somewhat puzzled as to the diagnosis, for my ideas before had been that purpura was associated with an endemic, debilitated, and in every respect a worn-out patient. But here I had a robust and healthy-looking individual, with no symptoms of bad dietary treatment. I made my diagnosis as stated above, which was in turn confirmed by Dr. Bronson. For treatment, I made the patient rest in bed, put him upon good dietary regime, and placed him upon half a teaspoonful of phosphate of soda three times a day. This latter I did because experiments seem to show that in these cases there is a lack of alkalinity in the blood. I kept him in the hospital for ten days, when he was discharged with only the remains of a pigmentation.

Purpura may be defined as a hemorrhage into the cutis arising idiopathically, and not due to traumatism. When the hemorrhages can be seen and yet there is no elevation of the surface, they are called petechise, in contradistinction to ecchymoses, where there is a swelling of the surface. Purpura is divided into the simple and hemorrhagic, the latter being only an exaggerated type of the former. Deering says that the simple forms of spantaneous hemorrhage often made their appearance suddenly at night, without any previous symptoms, and in adults the lower extremities are most frequently involved, chiefly on the flexor aspect of the thighs and calves, as occurred in the case reported. In the several forms of purpura, the hemorrhages may be so severe and profuse as to rapidly undermine the patient, and should they be in the meninges of the brain, to occasion rapid death. Hemorrhage at all times is severe, not in the proportion of how much, but the rapidity in which blood is lost. Purpura occurs in both sexes and all ages. From the various authorities I have consulted, I find the causes of the disease very numerous and often obscure. Among some of the etiological factors may be mentioned (1), many of the specific fevers, as typhoid, measles, etc.; (2) some drugs which seem to act especially upon the blood-vessels, as ergot, copaiba, quinine, etc.; (3) certain general diseases, as hæmaphilia, pernicious anæmia, etc.; (4) many diseases of the viscera, as the liver, spleen, etc.; (5) want of support to the blood-vessels, as in old age, and also functional nervous diseases are sometimes mentioned as a cause. Blood may escape into surrounding tissues, either by rupture of the vessel, diapedesis, or by transudation of the bood-coloring matter. In the case reported, three points could be seen where a distinct rupture had taken place, while the other lesions seemed dependent upon the transudation. Whenever there is an increase in blood pressure, there is transudation more likely to occur, as in stasis due to inflammation, or external pressure, or in the interruption of the blood current by an embolus or a thrombus. Again, changes in the vascular walls, by which they are weakened against the internal pressure, will afford grounds for transudation, as well as certain nervous changes, which play such an important part in the animal economy. Experience has shown that hemorrhages are more likely to occur in those parts most dependent, for the force of gravity is not confined exclusively to the inorganic world. In the case before us, the man was compelled to work all day upon his feet, and thus increased the blood pressure in those parts. Besides, he was a chronic bleeder, suffering from hæmaphilia, and to my mind, in those cases there is an insufficiency of the vascular walls, due either to want of development or to the lack of proper nourishment, as can often be seen by observing the growth of the economy at large. The liver of the patient was hyperæmic, and the functions somewhat deranged, which must have added fuel to the condition then present. The phosphate of soda I have always found to act well upon that organ, so that the treatment pursued afforded me a most excellent result.

---

IMPERFORATE VAGINA—CRANIOTOMY.—Dr. James B. Neal, of Tungchowfu, has reported a case that seems unique. A woman, 23 years of age, presented herself with a tumor in the abdomen, which was found to be a retained fœtus. The vagina was imperforate, resulting from ulceration. If the woman's story was correct, she had carried a dead child for three months past the time when she should have been delivered, when she had pains, but the native midwives denied that she was pregnant. After delivery, by section and craniotomy, she made rapid recovery.—Satellite, April, 1890.

---

A pupil in one of the public schools of the city complied recently in the following manner with a request to write a composition on the subject of a physiological lecture to which the school had just listened.

"The human body is made up of the head, the thorax, and the abdomen.

"The head contains the brains, when there is any.

"The thorax contains the heart and lungs.

"The abdomen contains the bowels, of which there are five: A, E, I, O and U, and sometimes W and Y."

